# Current News and Opinion

**Published:** 1885-11

**Authors:** 


### Current News and Opinion.

### FIRST DISTRICT DENTAL SOCIETY OF THE STATE OF NEW YORK.

OFFICERS, 1885–6.

Dr. William Carr, *President.*
Dr. J. F. P. Hodson, *Vice-President.*
Dr. B. C. Nash, *Secretary.*
Dr. Charles W. Miller, *Treasurer.*
Dr. J. B. Littig, *Librarian.*

EXECUTIVE COMMITTEE.

Dr. W. W. Walker, *Chairman,* Dr. A. L. Northrop, Dr. C. E. Francis.

CLINIC COMMITTEE.

Dr. C. F. W. Bodecker, *Chairman,* Dr. H. H. Atkinson, Dr. F. H. Lee.

BOARD OF CENSORS.

Dr. A. L. Northrop, *Chairman,* Dr. E. A. Bogue, Dr. W. A. Bronson, Dr. C. A. Woodward, Dr. Frank Abbott.

PAPERS TO BE READ BEFORE THE FIRST DISTRICT DENTAL SOCIETY OF THE STATE OF NEW YORK, 32D STREET AND BROADWAY, NEW YORK CITY, 1885–6.

Tuesday evening, May 5th, 1885—Dr. G. W. Weld, "Chloroform and the Fifth Pair of Nerves."

Tuesday evening, June 2d—C. E. Francis, D. D. S., M. D. S., "Cleansing Teeth."

Tuesday evening, September 22d—Wm. H. Atkinson, M. D., D. D. S., "Pyorrhœa Alveolaris."

Tuesday evening, October 6th—C. Heitzmann, M. D., "Microscopy as a Guide in Practical Dentistry."

Tuesday evening, November 3d—J. N. Farrar, M. D., D. D. S., "Mechanical Appliances for Correcting Irregularities of the Teeth." Blackboard Illustrations.

Tuesday evening, December 1st—Edwin T. Darby, M. D., D. D. S., Professor of Operative Dentistry and Dental Histology, University of Pennsylvania, "Erosion of the Teeth; its Cause and Treatment."

Tuesday evening, January 5th, 1886—S. H. Guilford, A. M, D. D. S., Professor of Operative and Prosthetic Dentistry, Philadelphia Dental College and Hospital of Oral Surgery, "The Band Matrix and its Uses."

Tuesday evening, February 2d—Frank Abbott, M. D., Professor of Operative Dentistry and Dental Therapeutics, New York College of Dentistry, "The Treatment of Pulpless Teeth."

Tuesday evening, March 2d—John J. R. Patrick, D. D. S., Bellville, Ill., "Progressive and Retrogressive Physiological Metamorphosis of the Jaws and Teeth."

Tuesday evening, April 6th—Election of Officers.

In addition to the regular meetings, a number of special meetings will be held, at dates fixed later, when clinics and papers will be given by the following gentlemen :

Clinic and Paper—Dr. Wm. Herbst, Bremen, Germany, "Inventor of Herbst Method of Filling Teeth."

Paper—M. Whillden Foster, M. D., D D. S., Professor of Dental Mechanism and Metallurgy, Baltimore College of Dental Surgery. "Abnormal Conditions of the Antrum and Restorative Treatment."

Clinic and Paper—Dr. H. C. Register, Philadelphia.

Clinic and Paper—Dr. E. Parmly Brown, Flushing, L. I. "New Porcelain Bridge and Crown Work."

A lecture on Facial Expression, by a very eminent Sculptor of New York City, illustrated by Clay Modeling, Prehistoric Skulls, etc., etc.

---

### FOREIGN BODIES IN THE ANTRUM OF HIGHMORE.

Dr. Mulhall presented three foreign bodies which he had removed from the antrum of Highmore of a lady, aged 23. Having diagnosed the case as one of purulent catarrh of the antrum of the right side, he sent her to a dentist with instructions that the first molar tooth be extracted and a gold tube be made with a clasp which would fit around the first bicuspid so as to keep it in place. She went to the dentist and had the tooth extracted, and he bored through the alveolus a proper opening into the antrum and washed out a quantity of fetid pus, but she stated that she did not think she could afford the tube. She went home and her husband undertook to make a tube that she could put into the antrum. She at once began to improve, and was getting along quite well under the use of carbolic acid and iodine washes, when the plug suddenly disappeared. Her husband a second time undertook to wash the antrum, and put in a hard rubber plug to keep the opening patulous till the next washing. Some two months after, this also disappeared. After the first six weeks the discharge had not been purulent, but when she got these foreign bodies in, the discharge again became offensive in character. As it was pretty evident that foreign bodies were in the antrum, he concluded to operate for their removal. The second molar had been removed, and so had the second bicuspid. First, separating the cheek from the jaw-bone as much as possible, avoiding the infra-orbital artery and cutting back as far as he could where the wall of the antrum is thinnest, and where there is most space to see into the cavity, he made, with a chisel, an incision about an inch long into the antrum above the alveolus and at right angles, far back. He then had an opening about as large as one's thumb. Introducing a pair of forceps he succeeded after some difficulty in removing a hard rubber plug. She said, "Now I know there are two more there, because that is the first one I put in." The doctor was able to see another one, which he removed with the forceps. Searching around for some time he finally found a dark object that he grasped with the forceps and finally succeeded in getting out. A drainage tube was introduced because he proposed to treat the cavity through the opening he had made. The other opening through the socket of the first molar has closed up. As soon as she recovers he will withdraw the drainage tube and allow the cheek to close the opening.—*Report of the Medico-Chirurgical Society in the St. Louis Courier of Medicine.*

---

### NEW ENGLAND DENTAL SOCIETY.

At the twenty-third annual meeting of the New England Dental Society, held in Burlington, Vermont, the following members were elected as officers for the ensuing year:

*President*—J. B. Coolidge, Boston, Mass.
*First Vice-President*—G. A. Young, Concord, N. H.
*Second Vice-President*—G. F. Waters, Boston, Mass.
*Secretary*—A. M. Dudley, Salem, Mass.
*Treasurer*—G. A. Gerry, Lowell, Mass.
*Librarian*—E. O. Kinsman, Cambridge, Mass.

A resolution was offered and passed that, in the opinion of the members, if an International Medical Congress with a section of Dental and Oral Surgery be not held in this country, an International Dental Congress should be called, and that the New England Dental Society pledges itself to heartily support the enterprise. The delegates elected to the next meeting of the American Dental Society were instructed to act in accordance with this resolution.

---

### NEW JERSEY STATE DENTAL SOCIETY.

The following members were elected as the officers for the ensuing year:

*President*—W. Pinney, Newark.
*Vice-President*—A. R. Eaton, Elizabeth.
*Secretary*—Chas. A. Meeker, Newark.
*Treasurer*—Geo. C. Brown, Elizabeth.
*State Prosecutor*—Chas. A. Meeker, Newark.

### BOARD OF EXAMINERS.

J. Hayhurst, Chas. W. Meloney, James G. Palmer, Frederick A. Levy.

### EXECUTIVE COMMITTEE.

W. Price Richards, Geo. E. Adams, G. Carleton Brown, B. F. Luckey.

### A PLAIN TRUTH WELL EXPRESSED.

A too liberal use of italics is an insult to the intelligence of the reader. A correspondent has a two-page article in the September number of —— — ——, in which there are no less than forty-two italicized words. Whether this is the fault of the writer or the editor, we do not know, but, at least, it displays a lack of confidence in the reader, to say nothing of the fact that such means are frequently employed to give prominence to certain parts, so that the reader will not notice the dearth of ideas.—*Salmagundi* (Dr. E. G. Betty) in *The Dental Register.*

---

### EVIDENCES OF PROSPERITY.

The Rumford Chemical Works, Providence, R. I., manufacturers of Prof. Horsford's Acid Phosphate, have recently purchased a commodious building and warehouse near their present location, where they propose to move their business a few months hence. This purchase has been necessitated by the demands of their large and increasing business, and it is pleasant to record such an evidence of well deserved success and prosperity.

---

### AN EXCELLENT INSTRUMENT.

Among the useful devices for facilitating dental operations that are worthy of especial mention, is the Ainsworth spring punch for making perforations in the rubber dam. It does the required work quickly, effectively, and it may be added, *perfectly.* It is almost indispensable to anyone who has used it, and every dentist should have one. The S. S. White Mfg. Co., make them. C. E. F.

---

### MINNESOTA.

The Minnesota State Board of Dental Examiners will meet in St. Paul, at the Ryan Hotel, Tuesday, the first day of Dec., 1885, for the purpose of examining candidates for admission to practice in the State, and to endorse diplomas. J. H. MARTINDALE,
Secretary.

---

### DELINQUENTS.

We earnestly request those of our subscribers who have not forwarded their subscription fee to do so without further delay. Postal notes are easily procured at the postoffices, which will be gladly received by our treasurer.

